# It’s Been on My Mind: Aging Veterans’ Daily Thoughts of Military Service, Rumination, and Negative Affect

**DOI:** 10.1093/geroni/igaf122.628

**Published:** 2025-12-31

**Authors:** Christina Marini, Katherine Fiori, Carly Lawrence, Amanda Emma

**Affiliations:** Adelphi University, Garden City, New York, United States; Adelphi University, Garden City, New York, United States; Adelphi University, Garden City, New York, United States; Adelphi University, Garden City, New York, United States

## Abstract

Aging veterans may be more likely to think about their prior military service due to age-related changes, such as bereavement and declining physical health. Such losses might trigger intrusive memories about one’s prior service, which may lead to distress (Davison et al., 2016). Veterans may also experience increased distress on days when they ruminate more about their current troubles/worries (Kashdan et al., 2012). This study therefore examined the degree to which veterans a) thought/talked about their prior service and b) ruminated about their current worries, and whether either was associated with their daily negative affect. The sample included 68 veterans (M age = 74.75, SD = 5.58) who completed a baseline questionnaire and up to 7 (and at least 4) end-of-day surveys. Most were male (95.6%), white/Caucasian (95.6%), married (92.6%), retired (79.4%), and served during the Vietnam era (79.4%). More than half (58.8%) completed at least one combat/warzone tour. After controlling for age and number of combat/warzone tours, findings from a series of multi-level models indicated that veterans who on average across days a) think/talk about their service more and b) ruminate more experience higher negative affect (i.e., between-person associations). Furthermore, a significant within-person association indicated that on days when veterans ruminate more than they usually do, they experience higher negative affect. Additionally, veterans who ruminate more on average tend to think/talk more about their service. These findings suggest that aging veterans’ affective well-being is tied to their active processing of both past service-related experiences and current worries.

